# Methodological and Statistical Considerations for the National Children's Study

**DOI:** 10.3389/fped.2021.595059

**Published:** 2021-08-20

**Authors:** Ron D. Hays, David Hubble, Frank Jenkins, Alexa Fraser, Beryl Carew

**Affiliations:** ^1^Division of General Internal Medicine, Department of Medicine, University of California, Los Angeles, Los Angeles, CA, United States; ^2^Westat Inc., Rockville, MD, United States

**Keywords:** reliability, validity, surveys, patient report, outcomes

## Abstract

The National Children's Study (NCS) statistics and item response theory group was tasked with promoting the quality of study measures and analysis. This paper provides an overview of six measurement and statistical considerations for the NCS: (1) Conceptual and Measurement Model; (2) Reliability; (3) Validity; (4) Measurement Invariance; (5) Interpretability of Scores; and (6) Burden of administration. The guidance was based primarily on recommendations of the International Society of Quality of Life Research.

## Introduction

### Methods

The National Children's Study (NCS) was an “integrated systems-based initiative to assess a full spectrum of health and capture the environmental factors and other influences that shape the trajectory of child development” (1). The NCS was designed to examine environmental factors associated with children's health and development and ultimately to improve the health of children. The Statistics and Item Response Theory Group facilitated the quality of measures developed for the NCS and worked to ensure adherence to high standards of measurement (Tables 1, 2). The group's guidance was based largely on recommendations of the International Society of Quality of Life Research (ISOQOL) (2).

**Table 1:** Key measure properties.

**Conceptual and measurement model** – The conceptual model provides a description and framework for the targeted construct(s) to be included in a Participant Reported Outcome (PRO) measure. The measurement model maps the individual items in the PRO measure to the conceptual construct
**Reliability** – The degree to which a PRO measure is free from random error.
**Internal consistency reliability** – The degree of the interrelatedness among the items in a multi-item PRO measure.
**Test-retest reliability** – A measure of the reproducibility of the scale, i.e., the ability to provide consistent scores over time in a stable population.
**Validity** – The degree to which a PRO instrument measures the PRO concept it purports to measure.
**Content validity** – The extent to which the PRO measure includes the most relevant and important aspects of a concept in the context of a given measurement application.
**Construct validity** – The degree to which scores on the PRO measure relate to other measures (e.g., participant- or patient-reported or clinical indicators) in a manner that is consistent with theoretically derived a priori hypotheses concerning the concepts that are being measured.
**Responsiveness** – The extent to which a PRO measure can detect changes in the construct being measured over time.
**Criterion validity** – The degree to which the scores of a PRO measure are an adequate reflection of a “gold standard.”
**Measurement invariance** – Evidence that the construct is generalizable across subgroups (e.g., gender, age)

**Table 2:** Recommendations for minimum standards for measures.

1	**Conceptual and measurement model** – A PRO measure should have documentation defining and describing the concept(s) included and the intended population(s) for use. In addition, there should be documentation of how the concept(s) are organized into a measurement model, including evidence for the dimensionality of the measure, how items relate to each measured concept, and the relationship among concepts included in the PRO measure.
2	**Reliability** – The reliability of a PRO measure should preferably be at or above 0.70 for group-level comparisons but may be lower if appropriately justified. A minimum reliability of 0.90 has been suggested for the use of measures at the individual level. Reliability can be estimated using a variety of methods including internal consistency reliability, test-retest reliability, or item response theory. Each method should be justified.
3	**Validity**
3a	**Content validity** – A PRO measure should have evidence supporting its content validity, including evidence that patients and experts consider the content of the PRO measure relevant and comprehensive for the concept, population, and aim of the measurement application. This includes documentation of: (1) qualitative and/or quantitative methods used to solicit and confirm attributes (i.e., concepts measured by the items) of the PRO relevant to the measurement application; (2) the characteristics of participants included in the evaluation (e.g., race/ethnicity, culture, age, gender, socio-economic status, literacy level) with an emphasis on similarities or differences with respect to the target population; and (3) justification for the recall period for the measurement application.
3b	**Construct validity** – A PRO measure should have evidence supporting its construct validity, including documentation of empirical findings that support predefined hypotheses on the expected associations among measures similar or dissimilar to the measured PRO.
3c	**Responsiveness** – A PRO measure for use in longitudinal research should have evidence of responsiveness, including empirical evidence of changes in scores consistent with predefined hypotheses regarding changes in the measured PRO in the target population for the research application.
4	**Interpretability of scores** – A PRO measure should have documentation to support interpretation of scores, including what low and high scores represent for the measured concept.
5	**Patient and investigator burden** – A PRO measure must not be overly burdensome for patients or investigators. The length of the PRO measure should be considered in the context of other PRO measures included in the assessment, the frequency of PRO data collection, and the characteristics of the study population. The literacy demand of the items in the PRO measure should usually be at a 6th grade education level or lower (i.e., 12-year-old or lower); however, it should be appropriately justified for the context of the proposed application

We developed a measurement template as a fillable form for Domain team members to provide information on the name of proposed measure, a summary description of the measure, the type of data collection for the measure, the measurement target, the respondent, the exposure or outcome measure, the age band of the measurement target, the mode of administration, the location of administration, the estimated time to administer, how the measure is scored, evidence for reliability and validity, whether the measure is an existing measure and if there are fees associated with use, an adaptation of an existing measure or a new measure, and special conditions for administration. The template was created to ensure consistency, avoid redundancy, and evaluate the quality of proposed items and instruments.

Guidance provided in the key aspects of measures document was intended to generate NCS measures that were psychometrically sound, practical (i.e., as parsimonious as possible), preferably free of intellectual property constraints (3), clear and easy to understand, and applicable across the lifespan (e.g., transition to adulthood). While the recommendations listed in Tables 1, 2 are targeted at outcome measures, they are generally applicable to all the proposed NCS measures. The implementation of common standards across a diverse and comprehensive set of exposure and outcome measures is a major strength of the study.

### Key Aspects of Measures

Measures are only valid if the participants in a study can understand what is being asked of them and can provide a response that accurately reflects their experiences, perspectives, abilities and/or levels of development. It is critical that questions and response options be clear and easy to understand. Qualitative testing of measures (e.g., cognitive interviews to evaluate if the wording of survey items are understood by respondents) should, whenever possible and appropriate, include individuals with a broad range of literacy and educational level. The key aspects of measures are: (1) Conceptual Framework and Measurement Model; (2) Reliability; (3) Validity; (4) Measurement Invariance; (5) Interpretability of Scores; and (6) Burden of administration.

### Conceptual Framework and Measurement Model

The first key property of a measure is the conceptual framework and measurement model for the construct to be measured and the mapping of items in the measure to the construct. This means there should be documentation of the concepts represented in a measure and the intended target of the measurement. In addition, evidence supporting the way elements of the measure are used to create scores need to be provided. That is, an instrument's “scoring strategy” (e.g., total scores, scale scores) needs to be empirically supported, whenever possible, through analyses such as hierarchical clustering (4, 5), or item-level factor analyses. Such analyses could include confirmatory factor analysis, but exploratory analyses could be conducted with the goal of identifying all sources of common variance, and local dependence violations that may impact scoring. It is important to note that the identification of multidimensionality, *per se*, does not necessarily vitiate the ability of a researcher to produce a meaningful scale score (6, 7).

For the NCS, conceptual and measurement models were to be included as feasible for all the measures.

### Reliability

The second key aspect of measurement is reliability, or the extent to which a measure yields a similar score when the target of the measure has not changed. Reliability can be estimated by using Cronbach's coefficient alpha (internal consistency reliability), test-retest reliability, and inter-rater reliability. Reliability and intraclass correlation formulas in terms of sources of variance (mean squares, MS) are provided in Table 3. The common element of reliability estimation is some form of replication of measurement. The reliability column provides estimates for the average of the replicated measures (e.g., multiple items assessing the same domain) while the intraclass correlation column provides estimates for a single measurement (e.g., for the “average” single item in a multiple-item scale, or the average rater).

**Table 3:** Intraclass correlation and reliability.

**Model**	**Reliability**	**Intraclass correlation**
One-way	$\frac{MS_{BMS}-MS_{WMS}}{MS_{BMS}}$	$\frac{MS_{BMS}-MS_{WMS}}{MS_{BMS}+\;{\left(k-1\right)MS}_{WMS}}$
Two-way mixed	$\frac{MS_{BMS}-MS_{EMS}}{MS_{BMS}}$	$\frac{MS_{BMS}-MS_{EMS}}{MS_{BMS}+\;{\left(k-1\right)MS}_{EMS}}$
Two-way random	$\frac{N(MS_{BMS}-MS_{EMS})}{NMS_{BMS}+\;MS_{JMS}-\;MS_{EMS}}$	$\frac{MS_{BMS}-MS_{EMS}}{MS_{BMS}+\;{\left(k-1\right)MS}_{EMS}+k\left(MS_{JMS}-MS_{EMS}\right)/N}$

*BMS, Between Ratee Mean Square; WMS, Within Mean Square; JMS, Item or Rater Mean Square; EMS, Ratee × Item (Rater) Mean Square; N, n of ratees; k, n of items or raters*.

Table 3 shows reliability and intraclass correlation formulas included in the Key Aspects of Measures document.

Internal consistency reliability is estimated using the two-way mixed model formula appearing in the reliability column of Table 3. That is, between- respondent variance is compared to the interaction between respondents and the items in a multiple-item scale. Note that concerns have been expressed regarding the reliance on alpha as an index of reliability (5, 8). One major concern is that alpha reflects the reliability of a unit-weighted composite and is influenced by all sources of common variance. Thus, if the measure is multidimensional, alpha will not reflect how well the scores reflect true standing on a single construct. In such cases, Revelle and Zinbarg (5) suggest indices such as coefficient omega hierarchical as an alternative to alpha. Researchers need to be clear about what their reported reliability indicates—the percent of reliable variance of a multidimensional composite, or the degree to which observed scores reflect true variation on a single construct. For uses in longitudinal measurement of the construct, test-retest reliability may be important to estimate, and normative standards for “normal variability” (including models of practice effects for ability assessments) will be important to provide so users can determine how much test- retest change is meaningful.

Test-retest reliability is typically estimated using the intraclass correlation column of the two-way fixed model. The intraclass correlation is the appropriate estimate because the reliability of concern is for a measure at a single point in time rather than the average of the measure at two-time points. In addition, the intraclass correlation readily generalizes to provide a single-number summary of consistency over time if the measure is administered on more than two occasions. Intra-rater reliability is estimated using the intraclass correlation column of the two-way random model if one is willing to assume that the raters are sampled from a population of raters rather than fixed. The one-way model row in Table 3 applies to situations when a higher-level unit such as a school or physician is the target of measurement and the source of information about these units are students or patients nested within the unit.

A minimum reliability of 0.70 is commonly accepted for group-level (as opposed to individual-level) use of measures (9). The standard error of measurement at this reliability level is ~0.55 of a standard deviation. A minimum reliability of 0.90 has been suggested for the use of measures at the individual (rather than group) level (9). At the 0.90 reliability level, the standard error of measurement is approximately one-third of a standard deviation. That means that the width of the 95% confidence interval around an individual's estimated true score is about 1.2 SD. If measures are scored as z- scores (mean of 0 and SD of 1) then the reliability = 1 − SE^2^. If measures are scored as T-scores (mean of 50 and SD of 10), then the reliability = 1 − (SE/10)^2^.

When item response theory is used to calibrate items in a measure, reliability is estimated conditional on the estimated score; rather than a single reliability estimate, the SE differs across respondents. And reliability is analogous to “information”: reliability = 1 − (1/information). Relatedly, the SE = 1/$\sqrt{Information{\;}^{\;}}$ and Information = 1/SE^2^. These reliability estimates are advantageous because measures are typically less reliable at the extreme ends of the scale.

Having the most accurate SE estimate for a given respondent is also important in identifying “responders” to interventions or classifying people over time into those who have stayed the same, gotten better, or gotten worse. The reliable change index (RCI) (10, 11) can be used to estimate whether there is significant change: Change/ ($\sqrt{{2\;}^{\;}}$ ^*^SEM), where SEM = standard error of measurement (SEM = SD^*^ $\sqrt{{1-reliability\;}^{\;}}$). An RCI of ≥ |1.96| is deemed to be statistically significant at *p* < 0.05. One can also identify significant individual change using the equivalent coefficient of repeatability (CR): (CR) = 2.77^*^SEM. If IRT standard errors are available, then the RCI can be estimated as: Change/$\sqrt{SE_{1}^{2}+SE_{2}^{2}}\;$(12).

We recommended assessment of reliability of instrument performance within the NCS environment. For example, the inter- rater reliability of a measure administered by NCS field workers could have been estimated, and the training and practice used to achieve that level of reliability documented and reported. While measures should have reliabilities of 0.70 or higher for group comparisons, it was expected that there would be some measures where reliabilities did not achieve this threshold. Larger standard errors associated with lower levels of reliability need to be considered when interpreting scores.

### Validity

The third key aspect of measurement is validity, or the extent to which the measure assesses what it is intended to measure (13, 14). There are multiple flavors of validity including content validity, and construct validity (including responsiveness and criterion validity). Content validity requires documentation of sources from which items were derived, modified, and prioritized during the measure development process. Qualitative methods allow instrument developers to capture patient perspectives on the concept and evaluate its comprehensiveness and acceptability. Individual interviewers or focus groups have been cited as “the preferred method for elucidating the patients' experiences” (15). Content expert review and content blueprints are often used to ensure content validity.

Construct validity means that there should be documentation of empirical findings that support predefined hypotheses on the expected associations among measures that are similar or dissimilar to the measure. The hypotheses should specify the direction and, whenever possible, the expected magnitude of the associations. Rules of thumb for the magnitude of correlations can be derived by translating effect sizes (d) into correlations. The Cohen effect size (d) rules of thumb are that 0.20 SD is small, 0.50 SD is medium, and 0.80 SD is large. r = d/$\sqrt{d^{2}+4}$. The corresponding correlations are therefore 0.100 (small), 0.243 (medium), and 0.371 (large). Whether this rule of thumbs works in any specific application is open to question. The context surrounding differences and correlations needs to be considered in interpreting the magnitude. Note that sample sizes of 386, 66 and 29 are needed for 0.100, 0.243 and 0.371 correlations, respectively, to be statistically significant at *p* < 0.05.

Just as the observed cross-sectional associations of a measure with other measures should correspond to a priori hypotheses, so should change over time for a measure (i.e., responsiveness to change). Responsiveness is an aspect of construct validity (16). External information (anchor) is used to indicate change and to see if the measure being evaluated is responsive to the change. Identifying anchors can be challenging but possibilities include natural changes (e.g., changes from injury) or experimentally- induced (e.g., pharmacologic interventions). Observed change on a measure is compared to the standard deviation at baseline (effect size), standard deviation of change (standardized response mean), or standard deviation of change for those deemed to be stable (responsiveness statistic). Estimation of the minimally important change for a measure is a special case of responsiveness to change because prospective change is examined for those deemed to have changed by a non-trivial but not large amount (16) that can be helpful for the interpretation of scores. Criterion validity is a subset of construct validity where the variables that are being compared with the target measure are deemed to be criteria or “gold standard” measures. In some situations, there may not be any existing criterion or legacy measures.

For the NCS we recommended that evidence be provided about the validity of the measures. Assessment of validity needs to consider performance within the NCS environment. For example, the NCS computer-based administration should be compared with the historic or original modes of assessment (e.g., paper and pencil) used to evaluate each measure to assess possible effects on validity.

### Measurement Invariance

A fundamental tenet of good measurement is that scores should mean the same thing across different subgroups. Ideally, items within a measure will display measurement invariance—individuals equal on the construct should have the same expected score regardless of their group membership (17). Evaluation of differential item functioning (DIF) is fundamental to evaluate measurement invariance (18). Measures of constructs that are ostensibly applicable across a wide age range present special challenges to longitudinal researchers. Because the indicators of the construct may change across age groups, measures of the same construct (e.g., aggression) may include different items at different age ranges. When this is the case, research needs to be conducted to demonstrate that the same construct is being assessed, and that scores are on the same scale (and thus comparable) across age groups. In IRT contexts, such research may involve “linking” studies where anchor items are then used to define a metric, and to then scale age-unique item sets onto an interpretable scale (19).

For the NCS, we recommended that evidence be provided about measurement equivalence for subgroups of the targeted population.

### Interpretability of Scores

The fifth key aspect of measurement is interpretability of scores. Measures need to be easily interpreted by different stakeholders including patients, clinicians, researchers, and policy makers. End-users must be able to know what a high or low score represents. In addition, knowing what comprises a meaningful difference or change in the score from one group to another (or one time to another) is needed to understand and interpret the measure. One way to enhance the interpretability of measures is to compare scores from a study to known scores in a population (e.g., the general US population or a disease subgroup). The availability of such benchmarks enhances understanding of how the study group scored compared to some reference or normative group. Also, subgroups of a sample can be compared (e.g., asymptomatic vs. mild vs. more severe disease). Understanding the nature of the questions being asked of the measurement process and the resulting nature of the normative standards being applied (e.g., census-based norms, age-corrected norms, norms corrected for all relevant demographic variables) is essential.

For the NCS we recommended that guidance be provided by subject matter experts and developers to field workers, participants, and potential analysts about the interpretability of scores.

### Burden

The sixth key aspect is the burden (patient and investigator) associated with administering the measure. All other things being equal, a more parsimonious measure is preferable especially when there are multiple measures being administered to subjects in a study. For self-administered paper and pencil surveys, about 3–5 items per minute can be administered (20). However, computer administration tends to result in quicker completion of survey items. For example, scleroderma patients were found to complete an average of 6 items per minute when completing PROMIS® items by computer (21).

For the NCS, field worker experience, subject matter expert advice, project officer assessments and other input was used to ensure tolerable respondent burden.

In sum, the evidence about the properties of proposed measures requires careful and thorough consideration. There is no single threshold for which an instrument is appropriate (e.g., valid vs. not valid) for all subgroups or applications. In addition, no single study can confirm all the measurement properties for all research contexts. Measurement advancement relies on the iterative accumulation of a body of evidence (maturation model) replicated in different settings. Thus, it is the weight of the evidence (i.e., number and quality of the studies and consistency of findings) that cumulatively informs the assessment of the appropriateness of a measure for any application.

## Data Availability Statement

The original contributions presented in the study are included in the article/supplementary material, further inquiries can be directed to the corresponding author.

## Author Contributions

RH drafted the articles. DH, FJ, AF, and BC provided edits. All authors contributed to the article and approved the submitted version.

## Conflict of Interest

DH, FJ, AF, and BC were employed by company Westat Inc. The remaining author declares that the research was conducted in the absence of any commercial or financial relationships that could be construed as a potential conflict of interest.

## Publisher's Note

All claims expressed in this article are solely those of the authors and do not necessarily represent those of their affiliated organizations, or those of the publisher, the editors and the reviewers. Any product that may be evaluated in this article, or claim that may be made by its manufacturer, is not guaranteed or endorsed by the publisher.
